# Mental health state and quality of life questionnaire in low vision assessment: a case report

**DOI:** 10.1186/1756-0500-7-667

**Published:** 2014-09-23

**Authors:** Rokiah Omar, Mohd Harimi Abd Rahman, Victor Feizal Knight, Mushawiahti Mustaphal, Zainora Mohammed

**Affiliations:** Optometry & Vision Science Program, School of Healthcare Sciences, Faculty of Health Sciences, Universiti Kebangsaan Malaysia, Jalan Raja Muda Abdul Aziz, 50300 Kuala Lumpur, Malaysia; Department of Ophtalmology, Faculty of Medicine, Universiti Kebangsaan Malaysia, Jalan Ya’cob Latif, Bandar Tun Razak, Cheras, 56000 Kuala Lumpur, Malaysia; Faculty of Medicine and Defence Health, National Defence University of Malaysia, Sungai Besi Camp, 57000 Kuala Lumpur, Malaysia

**Keywords:** Mental health, Quality of life, Low vision, Case history

## Abstract

**Background:**

Vision impairment associated with diabetic retinopathy, is well known and low vision rehabilitation is always recommended. In this report, the importance of objective measure of mental health and quality of life screening in diabetic retinopathy low-vision assessment is discussed.

**Case presentation:**

We describe the case of a 43-year-old Asian female who has mild vision impairment due to tractional retinal detachment secondary to diabetic retinopathy and how mental health screening and quality of life screening during low vision rehabilitation can improve in the management of this patient.

**Conclusion:**

Although vision impairment was mild, the psychological impact was enormous and affected her quality of life substantially. This case report illustrates that recognition of the mental health and quality of life impact on visual impairment is critical to the rehabilitation management of low vision patients with diabetic retinopathy.

## Background

Currently, routine low vision assessment normally involves the case history which includes the patient’s main complaint, the patient’s requirements, the patient’s ocular history, the patient’s general health plus medication, family history of ocular diseases and general health. The psychometric measurements such as visual acuity (VA), refraction, visual field assessment, contrast sensitivity function test, assessment of anterior and posterior part of the eyes are common examinations conducted during low vision assessment [1]. However, screening of the mental health state i.e. using The Depression Anxiety Stress Scales (DASS) [2] and Low Vision Quality of Life Questionnaires (LVQoL) [3] are not routine in case history taking during low vision assessment at our low vision clinic.

Mental health status can be objectively measured by DASS. DASS classifies mental health into depression, anxiety and stress categories. Each of these mental health problems is divided into mild, moderate, severe and very severe depending on the scores obtained from the questionnaires. The classification of DASS is shown in Table 1. Quality of life can be measure objectively using the LVQoL assessment questionnaire on a scale of 0–125, where 0 measures no quality of life and 125 measures the best achievable quality of life. The LVQoL score can be categorized to normal, mild, moderate and severe score and summarized in Table 2. The LVQoL is a reliable and fast method for measuring the quality of life among the visually impaired in a clinical setting. The LVQoL assessment questionnaire will also enable quantification of the quality of life of those with low vision pre- and post-low vision rehabilitation [3, 4].

**Table 1:** **Classification of the depression anxiety stress scales score for stress, anxiety and depression**

Category		Score	
	Stress	Anxiety	Depression
Normal	0-9	0-7	0-14
Mild	10-13	8-9	15-18
Moderate	14-20	10-14	19-25
Severe	21-27	15-19	26-33
Very severe	28+	20+	34+

**Table 2:** **Classification of low vision quality of life questionnaires score**

Level of low vision quality of life questionnaires	Score
Normal	94-125
Mild	63-93
Moderate	32-62
Severe	0-31

Low vision not only affects daily activities and quality of life but it has also been proved to have effect on mental health [5]. A prior study reported that patients with low vision experienced twice the depression, that the blind experienced and 5 times more depression compared with normal populations [6]. Anecdotal evidence from low vision specialists over the years noticed that about 1 out of 10 low vision patients attending our low vision clinic had some form of mental health problem. Furthermore, all low vision patients who attended the low vision clinic showed a general reduction in their quality of life. This was shown by their complaints of not being able to do their usual activities because of their reduced vision. Hence, it is important to determine the actual state of mental health and the level of quality of life objectively during low vision assessment. Using quantitative screening tools such as DASS and LVQoL questionnaires during low vision assessment will improve the management of low vision patients. A quantitative measurement will be more meaningful than subjectively asking the low vision patient of their feelings or their mental health state and their perceived quality of life.

## Case presentation

A 43-year-old Malay female was referred by an ophthalmologist to the low vision clinic for low vision rehabilitation assessment. She was diagnosed with tractional retinal detachment secondary to diabetic retinopathy. She reported that she was diagnosed with diabetes mellitus 5 years ago. Currently, the patient is on follow-up and receiving treatment for diabetes mellitus from a government hospital. Her treatment includes metformin 500 mg twice daily, glicazide 60 mg daily and insulin 18 unit/day (*nocte*). Her glycosylated hemoglobin (HbAIc) assay at her last visit was 7 mmol/L. Apart from the diabetes mellitus, she is also on treatment for hypertension with hydrochlorothiazide 12.5 mg daily and for cholesterol with atorvastatin 20 mg daily. However, there was no evidence of any end organ damage i.e. diabetic nephropathy or diabetic neuropathy. There was no family history of diabetes mellitus or retinal detachment. Her main complaints at presentation at the low vision clinic was 1) blurred vision at both distance and near but she had never used nor was ever prescribed with any contact lens or glasses, 2) difficulty in recognizing faces and 3) difficulty with orientation and mobility. She had exhausted all her efforts to get treatment/rehabilitation for her visual problem and was frequently told that “nothing more can be done” to alleviate her condition. She agreed to be referred to the low vision clinic by the private ophthalmologist as a last attempt to improve her vision and quality of life through low vision rehabilitation.

**Figure 1:**
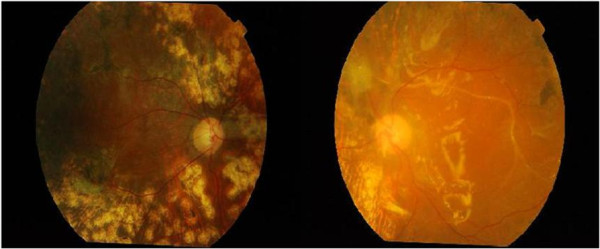
**Patient’s right eye and left eye showing the diabetic retinopathy changes.**

Upon examination, her distance vision for right eye (RE) and left eye (LE) was 6/48. Her near vision was N28 at 20 cm bilaterally. Subjective refraction, improved her distance vision to RE 6/48 using -1.75Ds, and LE 6/38 with a +3.50 Ds. For near, addition of +2.50 Ds enabled her to read N24 for RE and N16 for LE both at a reading distance of 20 cm. Visual field assessment testing at near using an Amsler’s chart revealed no abnormality. Contrast sensitivity function was not measured. Examination of her fundi using binocular indirect ophthalmoscopy and fundus photography showed diabetic retinopathy changes and tractional retinal detachment (Figure 1). She was informed of her eye condition with respect to the pathological process and its effect on her functional vision. The DASS score showed that she was in a state of severe stress (score = 34); she was severely anxious (score = 16), and she was depressed (score = 38). The LVQoL questionnaire assessment score was 43, which indicated that her quality of life was at the moderate stage.

Low vision assessment was conducted for near and distance vision. Using a +6.00 Ds spectacle magnifier she was able to improve her near vision to N10 at a reading distance of 20 cm. For her LE distance vision, a 3× monocular telescope was able to improve her vision to 6/12. She was also introduced to eccentric viewing techniques to identify/recognize faces. To assist her with orientation and mobility, a pair of distance spectacles with prescription of RE-1.75 Ds, LE +3.50 Ds and a 3x monocular telescope was prescribed. She was also referred to an orientation & mobility clinic at a training center organized by a blind person’s association. She agreed to a referral to the Social Welfare Department for registration purposes and eligibility assessment for benefits. To help her cope with her mental health status, she was referred to a clinical psychology clinic. She was also referred to an occupational therapy clinic to cope with her daily living activities. She was advised to return for a review at the low vision clinic in 3 months to monitor her functional vision for distance and near work and to repeat her DASS and LVQoL evaluations. Her mental health state will be monitored using DASS and her quality of life scores will be measured using the LVQoL questionnaire at the follow up.

After 3 months, the patient returned for a follow-up low vision. A routine low vision assessment was conducted and the DASS and LVQoL questionnaire was readministered. It was found that the patient’s visual acuity for both eyes remains the same i.e. 6/48. Subjective refraction and visual field status were similarly unchanged. The DASS and LVQoL questionnaire on the other hand showed significant improvement. The DASS score indicated that the stress level had come down from extremely severe (score = 34) to normal (score = 14) and the depression level also registered a decrease from extremely severe to moderate (score = 18) level. The anxiety score remained at 16 while the LVQoL score markedly improved from 43 to 98. A further three months review was given to the patient at the low vision clinic. At the same time, the patient was advised to continue with her sessions at the psychology clinic, occupational therapy clinic and the orientation & mobility clinic at the association for the blind.

## Discussion and conclusion

The visual impairment suffered by this low vision patient is considered relatively mild and capable of improvement using prescriptions of spectacles and low vision aids. However, the impact of the vision impairment to the mental health status and the quality of life was profound. These factors were identified during the low vision assessment by conducting deliberate screening of her mental health status and her quality of life using DASS and LVQoL respectively. The screening of the mental health results showed that she was experiencing severe stress, moderate anxiety and extremely severe depression. These findings were in agreement with previous studies that showed that low vision patients were more likely to suffer from depression as compared to normal populations [6, 7]. These objective findings alerted the examiner to refer this patient to clinical psychologist for further treatment as part of the management of low vision rehabilitation. The LVQoL scores also indicated that this patient was experiencing only a moderate quality of life. Again this objective finding alerted the examiner to refer this low vision patient to the occupational therapist for further assistance in coping with her daily living activities as part of the low vision management. Normally, the mental health status and quality of life assessment are not taken into account in routine low vision assessment and follow up at our low vision clinic. This case study showed that screening of mental health using DASS and LVQoL questionnaires is very important and should be incorporated in the overall management plan of low vision patients.

From this case study, it was also noted that even with mild vision Impairment, the low vision patient experienced multiple types of mental health problems with varying degrees of severity. The mental health status can affect the acceptance levels of the low vision patient towards rehabilitation. Therefore, in planning vision rehabilitation for the low vision patient, it is important that the examiner be aware of these factors so that so that an appropriate approach can be administered to the patient. Previous studies have shown that different states of mental health can affect the rehabilitation process [8]. Where low vision rehabilitation has failed, the failure may have possibly been due to the multiple mental health problems faced by the low vision patient, which were not identified and therefore not addressed. Consequently the level of acceptance for vision rehabilitation can become low. Thus it is important to obtain mental health status information during case history in low vision assessment. Working closely with clinical psychologists is also very crucial to ensure success in the management of low vision patients especially through a multidisciplinary approach.

The objective assessment of quality of life is an important aspect of low vision rehabilitation, which should be taken into account because visual impairment will affect the quality of life of the low vision patient [9–11]. In low vision management, the intervention offered is very task specific. Therefore identifying the low vision patient’s needs is very important. From this case study, it was found that the patient’s quality of life was reduced despite having only mild visual impairment. When quality of life declines, the patient’s motivation to participate in daily activities and social activities will also reduce [12]. Subsequently, the low vision patient tends to withdraw from families, friends and society. This can eventually accelerate further mental health problems [5]. Hence, it will make low vision rehabilitation more difficult to be successful. However when information on the quality of life status is known appropriate interventions and coping strategies can be structured to improve daily activities. These findings again encouraged a multi-disciplinary approach to the management of the low vision patient.

## Conclusion

This case study showed that screening of mental health using DASS and LVQoL quality of life questionnaires during low vision assessment can provide extra information about the low vision patient during case history. Objective information gathered from DASS and LVQoL can be used by the attending optometrist for a better plan in low vision rehabilitation. A series of studies or clinical trials should be explored to enhance the findings of this case study. Furthermore, the information gathered through DASS and LVQoL will encourage a multiple-disciplinary approach to the management of low vision patients. By conducting screening of mental health and quality of life questionnaires, early intervention can be instituted as the need arises. Therefore, it is recommended that the DASS and LVQoL be implemented during case history when low vision assessment is performed.

## Consent

Written informed consent was obtained from the patient for publication of this case report and accompanying images. A copy of the written consent is available for review by the Editor-in-Chief of this journal.
